# How multisystem inflammatory syndrome in children discriminated from Kawasaki disease: a differentiating score based on an inception cohort study

**DOI:** 10.1007/s10067-022-06444-0

**Published:** 2022-11-21

**Authors:** Ali Sobh, Doaa Mosad Mosa, Nada Khaled, Mai S. Korkor, Mohamed Ahmed Noureldin, Ahmad M. Eita, Marwa H. Elnagdy, Mohammed A. El-Bayoumi

**Affiliations:** 1 Department of Pediatrics, Mansoura University Children’s Hospital, Mansoura University Faculty of Medicine, Mansoura, Egypt; 2 Rheumatology & Rehabilitation Department, Mansoura University Hospitals, Mansoura University Faculty of medicine , 60 Elgomhoria St, Mansoura, 35516 Egypt; 3 Department of Clinical Pathology (Hematology Unit), Faculty of Medicine, Mansoura University, Mansoura, Egypt; 4 Department of Pediatrics & Neonatology, Horus University, Mansoura, Egypt; 5 Department of Medical Biochemistry and Molecular Biology, Faculty of Medicine, Mansoura University, Mansoura, Egypt

**Keywords:** Comparison, Discrimination score, Kawasaki disease, Multisystem inflammatory syndrome in children

## Abstract

**Background:**

About 25–50% of multisystem inflammatory syndrome in children (MIS-C) patients meet the criteria for diagnosis of Kawasaki disease (KD). The differentiation of both conditions is so challenging on clinical practice as the management of both is time dependant and precise diagnosis is fundamental.

**Method:**

Data were collected from children < 18 years old hospitalized with MIS-C or KD. Patient demographics, clinical, and laboratory data were compared, and a discrimination score was created to assist in clinical differentiation.

**Results:**

72 patients with MIS-C and 18 with KD were included in the study. Patients with MIS-C had a higher prevalence of abdominal pain (*p* = 0.02), vomiting (*p* = 0.03), and cervical lymphadenopathy (*p* = 0.02) compared with KD cases. MIS-C patients had higher liver enzymes (aspartate aminotransferase (AST) (*p* = 0.04), alanine aminotransferase (ALT) (*p* = 0.03), serum creatinine (*p* = 0.03), and lower platelet count nadir (*p* = 0.02) than KD. Four variables were detected in the regression analysis model, and the independent predictors were utilized to generate a scoring model that distinguished MIS-C from KD with an area under the curve of 0.70.

**Conclusion:**

This study constructed a prediction model for differentiation of MIS-C from KD based on clinical and laboratory profiles. This model will be valuable to guide clinicians in the treatment decisions.

**Table:** 

**Key Points** *• Children with MIS-C are more likely to have gastrointestinal symptoms, cervical lymphadenopathy, and respiratory involvement than KD patients.* *• Elevated liver enzymes and lower platelet count are more pronounced laboratory findings in MIS-C than KD.* *• This study constructed a prediction model for differentiation of MIS-C from KD based on clinical and laboratory profiles. This model will be valuable to guide clinicians in the treatment decisions.*

## Introduction

As the COVID-19 pandemic had spread across the world, a novel hyperinflammatory state dubbed as multisystem inflammatory syndrome in children (MIS-C) had emerged. MIS-C is an immune activation syndrome but not an acute infectious process, and patients do not currently have active infection [1].

The main issue is that about 25–50% of MIS-C patients meet the criteria for diagnosis of Kawasaki disease (KD) and resembles its presentation. Furthermore, there was no evidence of prior severe acute respiratory syndrome coronavirus-2 (SARS-CoV-2) exposure in these patients; hence, it might be so difficult to discriminate them from those with KD [2]. The differentiation of both conditions is so challenging on clinical practice as the clinical management of both is time dependant and precise diagnosis is fundamental [3, 4].

According to the center for disease control and prevention (CDC), MIS-C is defined as an individual < 21 years with fever, laboratory evidence of inflammation, with multisystem (≥ 2) organ involvement (cardiac, mucocutaneous, respiratory, hematologic), and no alternative diagnoses. In addition to the positive tests for SARS-CoV-2 infection by polymerase chain reactant (PCR), serology, antigen test; or COVID-19 exposure within the 4 weeks prior to the onset of symptoms [5].

Meanwhile, KD predominantly affects children between 6 months and 5 years of age. The prevalence is higher in children of Asian descent. It is a disease of 5 days fever or more and at least 4 of the following: rash, cervical lymphadenopathy, bilateral conjunctival injection, oral mucosal changes, and peripheral extremity changes [6].

This hyperinflammatory state and KD are overlapping in many clinical presentations such as high fever, diffuse skin rashes, mucous membrane changes, conjunctivitis, extremities erythema/edema, and cervical lymphadenopathy [7–9]. However, apparent differences in clinical and laboratory findings have been observed between the two syndromes. For example, gastrointestinal (GI) symptoms (vomiting, abdominal pain, or diarrhea) and neurological complications are more noticeable in MIS-C [10–12].

Cardiac presentations of the two diseases are quite diverse, the main complication of KD is coronary artery aneurysms in < 10% of cases [13, 14]. By contrast, coronary artery dilation in MIS-C has an incidence rate of 14–36% [15, 16]. Meanwhile, the most common cardiovascular complications in MIS-C are ventricular dysfunction, myocarditis, and pericardial effusion. Besides, 33–87% of MIS-C patients experienced shock and hemodynamic instability that required inotropic support, while 2–7% of KD children developed shock [17, 18].

Pulmonary symptoms are usually absent in KD, but in MIS-C, respiratory involvement usually consists of respiratory insufficiency, pleural effusions, and pulmonary infiltrates [19, 20]. In addition, hypercoagulable state occurs in both entities but is more pronounced in MIS-C [10, 21].

Regarding the dissimilarities in laboratory features of MIS‑C and KD, the inflammatory marker elevation observed in KD cases is moderate compared to MIS-C [22, 23]. Moreover, lymphopenia and thrombocytopenia are present in 37–81% of MIS-C patients [19, 24, 25], whereas they are rare in KD. Another characteristic of MIS-C is a marked increase in the biochemical markers of cardiac injury such as troponin T and B-type natriuretic peptide [4, 26].

Studies of both KD and MIS-C about the disease immunopathogenesis showed increased interferon-γ (IFN-γ)-associated cytokines, interleukin-1 (IL-1), IL-6, IL-18, and tumor necrosis factor (TNF) signaling pathways [7, 27, 28]. However, the subtle difference between MIS-C and KD presentation might be related to the different pathogenesis of the two diseases; compared with MIS-C patients, IL-1, IL-17A, and anti-endothelial cell antibodies are significantly higher in KD with the lacking of polyclonal proliferation of TCR Vβ 21.3 + activated CD4 + and CD8 + T cells [29, 30].

The distinction between MIS-C from KD can be challenging for health care practitioners, as prompt recognition of both patients is crucial for appropriate treatment. The current study underpins the demographic, clinical, and laboratory differences between both conditions that can assist in establishing the diagnosis and optimal management. In addition to a simple scoring model that will be valuable for distinguishing MIS-C from KD and guide clinicians decisions in future studies.

## Patients and method

### Subjects

This an inception cohort study was conducted on a group of children who attended to Mansoura University Children’s Hospital (MUCH) from January 2020 to December 2021.

**Fig. 1:**
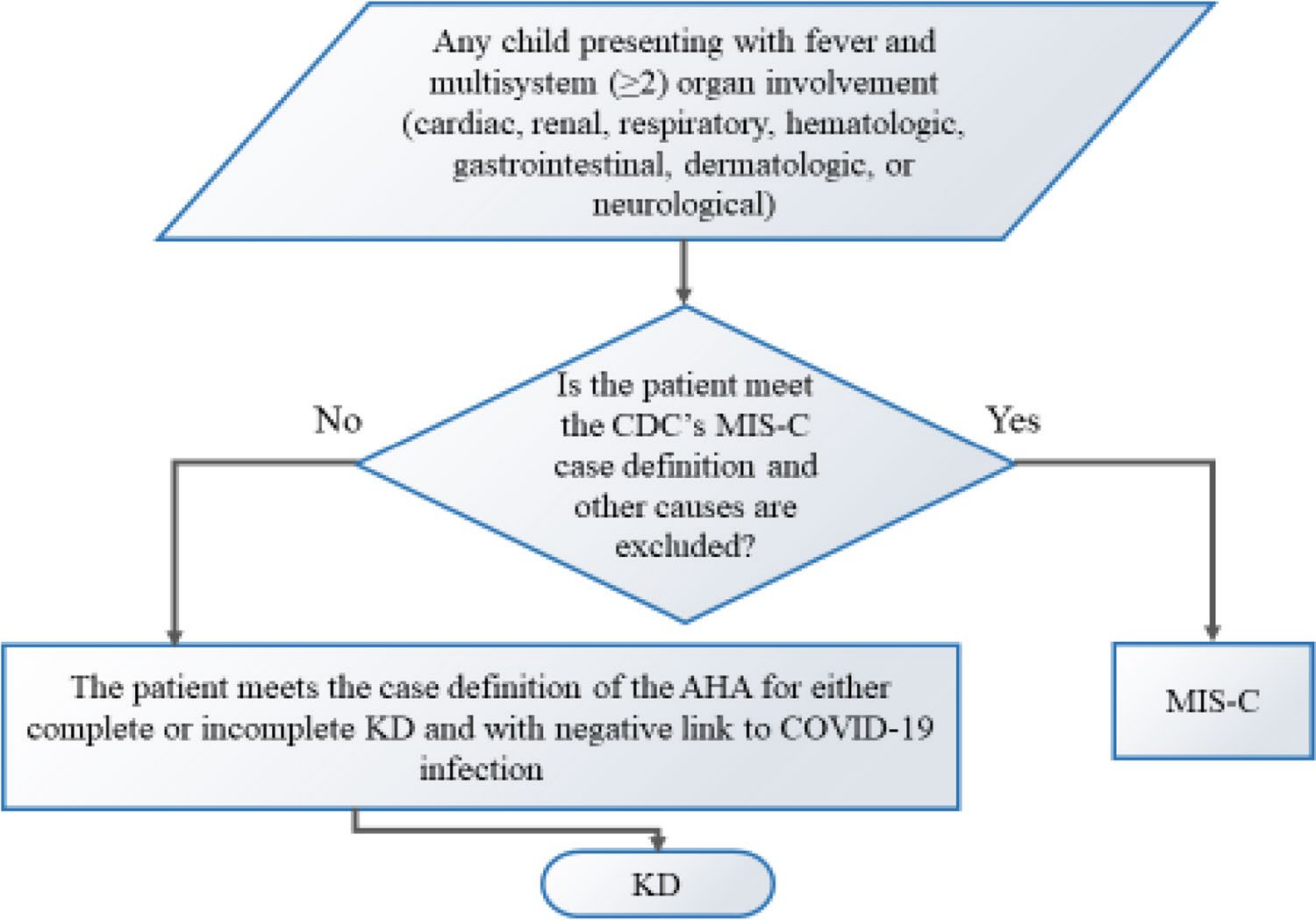
Flow chart of the included cases selection in the study

Inclusion criteria: (Fig. 1).

1. All children with a diagnosis of either complete or incomplete KD according to the American Heart Association criteria (AHA) diagnostic criteria [6] with negative link to COVID-19 infection (negative SARS-CoV-2 PCR, serology, antigen test, and negative contact history).
2. All children with CDC’s MIS-C case definition [5].
3. Patient whose parents signed a consent to be included in the study.

Exclusion criteria:

Patients who did not fulfil any of the above-mentioned definitions were excluded from the study.

### Data collection

Data were collected from our medical files and interpreted with respect to the followings:

1. Demographic: age, sex, contact history, and duration of symptoms before admission in days.
2. Clinical features and course: fever, GI, central nervous system (CNS), respiratory, cardiovascular, or mucocutaneous involvement. Besides, disease complications, intensive care unit (ICU) admission, its duration, treatments details (anti-platelets, intravenous immunoglobulins (IVIG), steroids), and outcomes.
3. Laboratory findings at the peak of the disease: complete blood count (CBC), erythrocyte sedimentation rate (ESR), C-reactive protein (CRP), alanine aminotransferase (ALT), aspartate aminotransferase (AST), serum ferritin, serum creatinine, D-dimer, SARS-CoV-2 PCR, and SARS-CoV-2 antibody test. However, cardiac biomarkers were not routinely done for all cases so no highlights about them in this article.
4. In addition to imaging, such as echocardiography (ECHO) and high resolution computed tomography (HRCT) chest.

### Statistical analysis

Data were analyzed using the IBM-Statistical Package of Social Science (SPSS) program for Windows (version 22). The normality of data was first tested. Qualitative data were described using numbers and percentages. Association between categorical variables was tested using the chi-square test, while the Fischer exact test was used when the expected cell count less than 5. Continuous variables were presented as mean ± SD for normally distributed data and median (min–max) for non-normal data. The two groups were compared with the Student *t*-test for normal data and the Mann Whitney test for non-normal data. The ability of each variable to differentiate MIS-C from KD was estimated with sensitivity, specificity analysis, and the best cut-off detection of continuous variables.

Significant variables entered into the logistic regression model to predict the most significant determinants and to control for possible interactions and confounding effects. Odds ratio (OR) were calculated with 95% confidence intervals. Sensitivity and specificity at different cut-off points were tested by the receiver operating characteristic (ROC) curve. Besides, the estimated probability of a child to have MIS-C versus KD based on the weighted regression results was calculated at the score and cut-off points. For all the above-mentioned statistical tests, the results were considered significant when *p* ≤ 0.05.

## 3-Results

A total of 90 patients were included of which 72 cases (80%) with MIS-C, 18 children (20%) with KD. Table 1 exhibits a description of demographics, clinical features, and outcomes of both categories. History of contact with probable or confirmed COVID-19 cases was negative in 60% of MIS-C cases. Among patients with MIS-C, 65.3% were male, whereas they represented 77.8% of cases in KD group. Most of MIS-C cases were older than 6 years old (54.2%). About 40.3% MIS-C children were admitted to ICU, which was higher than for KD (5.6%; *p* = 0.005). Patients with MIS-C generally had longer median ICU days (7 days) compared with patients with KD (5 days; *p* < 0.015). Twenty-one patients died in MIS-C cases (29%), but no deaths among patients with KD. The most frequently reported clinical findings were fever (90%), vomiting (50%), and skin rash (44.4%) in both groups.

**Table 1:** Comparison of clinical characteristics of MIS-C and Kawasaki disease

	Total *n* = 90	MIS-C *n* = 72 (80%)	KD *n* = 18 (20%)	Test of significance
Age/years < 6 ≥ 6	42 48	33 (45.8) 39 (54.2)	9 (50.0) 9 (50.0)	χ^2^ = 0.100 *p* = 0.751
Sex Male Female	61 29	47 (65.3) 25 (34.7)	14 (77.8) 4 (22.2)	FET = 1.03 *p* = 0.310
Contact history No Yes	47 43	29 (40.3) 43 (59.7)	18 (100) 0	χ^2^ = 20.58 *p* < 0.001*
Duration of symptoms before admission (days) Median (IQR)		5 (5–8)	6 (5–9)	*z* = 0.780 *p* = 0.435
Duration of hospital stay (days) Median (IQR)		8 (4–11)	7 (5–9.5)	*z* = 0.319 *p* = 0.749
Inpatient outpatient	46 44	33 (45.8) 39 (54.2)	13 (72.2) 5 (27.8)	χ^2^ = 4.01 p = 0.045*
ICU admission No Yes	60 30	43 (59.7) 29 (40.3)	13 (94.4) 1 (5.6)	χ^2^ = 7.81 *p* = 0.005*
ICU duration (days) Median (IQR)		7 (4.5–10)	5 (5–5)	*z* = 2.42 *p* = 0.015*
Outcomes Alive Died	69 21	51 (70.8) 21 (29.2)	18 (100) 0	χ^2^ = 6.85 *p* = 0.009*
Fever	81	63 (87.5)	18 (100)	χ^2^ = 2.50 *p* = 0.114
Respiratory tract symptoms: Runny nose	13	13 (81.1)	0	χ^2^ = 3.79 *p* = 0.051
Sore throat	11	9 (12.5)	2 (11.1)	χ^2^ = 0.026 *p* = 0.872
Anosmia	1	1 (1.4)	0	FET *p* = 0.362
Cough	31	28 (38.9)	3 (16.7)	χ^2^ = 3.15 *p* = 0.076
Dyspnea	23	22 (30.6)	1 (5.6)	χ^2^ = 4.73 *p* = 0.03*
Gastrointestinal symptoms: Diarrhea	20	15 (20.8)	2 (11.1)	χ^2^ = 0.402 *p* = 0.526
Abdominal pain	31	29 (40.3)	2 (11.1)	χ^2^ = 5.43 *p* = 0.02*
Vomiting	45	40 (55.6)	5 (27.8)	χ^2^ = 4.44 *p* = 0.035*
Neurological symptoms: Headache	5	5 (6.9)	0	χ^2^ = 1.32 *p* = 0.250
Seizures	4	4 (5.6)	0	FET = 1.05 *p* = 0.580
Disturbed consciousness	12	12 (16.7)	0	χ^2^ = 3.46 *p* = 0.063
Skin rash	40	29 (40.3)	11 (66.6)	χ^2^ = 2.53 *p* = 0.112
Respiratory signs: Cyanosis	8	8 (11.1)	0	χ^2^ = 2.19 *p* = 0.138
Hypoxemia	13	13 (33.3)	0	χ^2^ = 2.37 *p* = 0.124
Respiratory distress grades: 0 1 3 4	65 16 1 8	48 (66.7) 15 (20.8) 1 (1.4) 8 (11.1)	17 (94.4) 1 (5.6) 0 0	MC *p* = 0.128
Crepitation	15	15 (20.8)	0	χ^2^ = 4.50 *p* = 0.034*
Wheezes	5	5 (6.9)	0	χ^2^ = 1.32 *p* = 0.250
Pneumonia	65	58 (80)	4 (22.2)	χ^2^ = 12.46 *p* < 0.001*
CT chest CO-RAD 1 2 3 4 5	*n* = 50 13 14 7 6 10	12 (25.5) 13 (72.7) 6 (12.8) 6 (12.8) 10 (21.3)	1 (33.3) 1 (33.3) 1 (33.3) 0 0	MC = 1.97 *p* = 0.741
Cervical lymphadenopathy	14	12 (16.6)	2 (11.1)	χ^2^ = 5.41 *p* = 0.02*
Cardiac involvement Myocardial dysfunction Coronary artery dilation Valve involvement Shock/hypotension	*n* = 17 8 8 1 12	8 (11.1) 6 (8.3) 1 (1.4) 12 (16.7)	0 2 (11.1) 0 0	χ^2^ = 2.54 *p* = 0.468 FET = 3.46
AKI	17	15 (20.8)	2 (11.1)	χ^2^ = 0.732 *p* = 0.392
KIDGO stages 1 2 3	*n* = 17 4 7 5	3 (26.7) 6 (40) 5 (33.3)	1 (50) 1 (50) 0	MC = 1.04 *p* = 0.596
Treatment options IVIG IVIG + anti-platelet IVIG + anti-platelet + steroid		15 (28.3) 26 (49.1) 12 (22.6)	0 17 (100) 0	χ^2^ = 14.09 *p* = 0.001*
Need for vasoactive drug	31	30 (41.7)	1 (5.6)	χ^2^ = 8.32

*MIS-C* multisystem inflammatory syndrome in children, *KD* Kawasaki disease, *IQR* interquartile range, *ICU* intensive care unit, *CO-RAD* COVID-19 Reporting and Data System, *AKI* acute kidney injury, *KIDGO* Kidney Disease Improving Global Outcomes, *IVIG* intravenous immunoglobulins

^*^P value ≤ 0.05 is considered statistically significant

Compared with KD patients, cases with MIS-C were more likely to have GI symptoms such as abdominal pain (40.3% vs. 11.1%; *p* = 0.02), vomiting (55.6% vs. 27.8%; *p* = 0.035), cervical lymphadenopathy (16.6% vs. 11.1%; *p* = 0.02). Moreover, patients with MIS-C had a higher prevalence of respiratory manifestations, including dyspnea (30.6% vs. 5.6%; *p* = 0.03), crepitation (20.8%; *p* = 0.034), and pneumonia (80% vs. 22.2%; *p* < 0.001). Other respiratory clinical manifestations (runny nose, sore throat, anosmia, and cough) or respiratory signs (cyanosis, hypoxemia, respiratory distress, wheezes, and CT chest abnormalities) were more frequently reported in MIS-C cases rather than KD patients.

Diarrhea, cardiovascular (myocardial dysfunction, coronary artery dilation, valve involvement, and shock/hypotension), neurologic (headache, seizures, and disturbed consciousness), or renal presentations were more likely to occur in MIS-C patients compared to KD but did not attain a statistically significant difference. Management options for each group were displayed at the end of Table 1.

A summary of laboratory difference between KD and MIS-C is shown in Table 2. On comparison with KD, patients with MIS-C had higher liver enzymes (AST (*p* = 0.04), ALT (*p* = 0.038)), serum creatinine (*p* = 0.036), and lower platelet count nadir (*p* = 0.025). Despite of lower lymphocyte count, higher peak CRP, and serum ferritin in MIS-C patients than KD, the difference did not reach a statistical significance. Analysis of sensitivity, specificity, negative predictive value, positive predictive value, and accuracy of each significant parameter was shown in Table 3.

**Table 2:** Comparison of laboratory features of MIS-C and Kawasaki disease

	Total *n* = 90	MIS-C *n* = 72 (80%)	KD *n* = 18 (20%)	Test of significance
SARS-CoV-2 IgM	39	39 (51.4)	0	χ^2^ = 9.51 *p* = 0.002*
SARS-CoV-2 IgG	41	41 (54.2)	0	χ^2^ = 18.83 *p* < 0.001*
SARS-CoV-2 PCR	28	28 (38.8)	0	χ^2^ = 4.19 *p* = 0.04*
Leucocyte count nadir (10^3^/μL) Median (IQR)		10.17 (7.64–15.32)	10 (6.31–13.02)	*z* = 0.537 *p* = 0.591
Hemoglobin nadir (g/dl) Mean ± SD		10.63 ± 3.75	9.78 ± 1.44	*z* = 1.01 *p* = 0.314
Platelet nadir (10^3^/μL) Median (IQR)		286.8 (132.25–438.08)	502 (221.25–647.35)	*z* = 2.25 *p* = 0.025*
Lymphocytes nadir (10^3^/μL) Median (IQR)		2.5 (1.6–4.8)	3.25 (2.23–12.15)	*z* = 0.737 *p* = 0.461
Peak neutrophils (10^3^/μL) Median (IQR)		9.31 (5.70–14.63)	8.53 (5.56–12.4)	*z* = 0.348 *p* = 0.728
Peak ferritin (ng/ml) Median (IQR)		334 (155–700)	323.45 (130.38–722.58)	*z* = 0.303 *p* = 0.762
Peak ALT (IU/L) Median (IQR)		48.5 (21–150.25)	19 (17–40)	*z* = 2.08 *p* = 0.038*
Peak AST (IU/L) Median (IQR)		39 (26–135.25)	33 (22–46.5)	*z* = 2.04 *p* = 0.04*
Peak creatinine (mg/dl) Median (IQR)		0.65 (0.5–1.38)	0.5 (0.45–0.60)	*z* = 2.096 *p* = 0.036*
Peak Na (mmol/L) Mean ± SD		141.09 ± 20.50	135.97 ± 7.08	*t* = 0.604 *p* = 0.549
Peak CRP (mg/L) Median (IQR)		103.5 (38.75–171.75)	78 (47–165)	*z* = 0.007 *p* = 0.994
D-dimer (ng/ml) Median (IQR)		1.98 (1.3–5)	2 (2–2)	*z* = 0.217 *p* = 0.828
CRP levels > 99.5 (mg/L) No Yes	53 37	41 (56.9) 31 (43.1)	12 (66.7) 6 (33.3)	χ^2^ = 0.562 *p* = 0.453
Leucocyte count Normal Leukopenia Leukocytosis	44 1 45	34 (47.2) 1 (1.4) 37 (51.4)	10 (55.6) 0 8 (44.4)	χ^2^ = 0.593 *p* = 0.743
Platelet count Normal Thrombocytopenia Thrombocytosis	57 25 8	44 (63.3) 22 (30.6) 6 (8.3)	13 (72.2) 3 (16.7) 2 (11.1)	χ^2MC^ = 1.41 *p* = 0.495

*MIS-C* multisystem inflammatory syndrome in children, *KD* Kawasaki disease, *SARS-CoV-2* severe acute respiratory syndrome coronavirus-2, *PCR* polymerase chain reaction, *IQR* interquartile range, *ALT* alanine aminotransferase, *AST* aspartate aminotransferase, *Na* sodium, *CRP* C–reactive protein

^*^P value ≤ 0.05 is considered statistically significant

**Table 3:** The sensitivity and specificity analysis of clinical and laboratory predictors of MIS-C

	Sensitivity% (95%CI)	Specificity% (95%CI)	PPV% (95%CI)	NPV% (95%CI)	Accuracy% (95%CI)
Dyspnea	94.4 (72.7–99.8)	30.6 (20.2–42.5)	25.4 (15.8–37.7)	95.7 (76.0–99.8)	43.3 (36.3–57.7)
Abdominal pain	88.9 (65.3–98.6)	40.3 (28.9–52.5)	27.1 (16.7–40.4)	93.5 (77.2–98.9)	50.0 (46.1–67.4)
Vomiting	72.2 (46.5–90.3)	55.6 (43.4–67.3)	88.9 (75.2–95.8)	28.9 (16.8–44.5)	58.9 (53.08–73.75)
Crepitation	100 (81.5–100)	20.8 (12.2–32.02)	100 (0.746–1.0)	24 (15.2–35.5)	36.7 (24.4–44.8)
Cervical lymphadenopathy	88.9 (79.3–95.1)	33.3 (13.3–59.01)	84.2 (73.6–91.2)	42.9 (18.8–70.4)	77.8 (70.5–87.88)
Platelet nadir (10^3^/μL)	92.9 (72.7–99.8)	41.4 (28.9–52.5)	24.1 (17.8–41.6)	96.7 (80.9–99.8)	50.0 (47.4–68.62)

*NPV* negative predictive value, *PPV* positive predictive value

Four variables were detected in the univariate logistic analysis including abdominal pain, vomiting, cervical lymphadenopathy, and platelet nadir, which were the significant predictors of MIS-C from KD. However, on multivariate analysis, vomiting and cervical lymphadenopathy were detected as the independent factors for discriminating MIS-C from KD (Table 4).

**Table 4:** Regression analysis for independent predictors of MIS-C from Kawasaki disease

	Univariate analysis		Multivariate analysis		
	*P* value	COR	*β*	*P* value	AOR
Dyspnea	0.058	7.48 (0.936–59.77)			
Abdominal pain	0.032*	5.39 (1.15–25.26)	1.11	0.192	3.03 (0.573–16.06)
Vomiting	0.041*	3.25 (1.05–10.07)	1.71	0.042*	5.52 (1.07–28.49)
Crepitation	0.998	UNDEFINED	-	-	
Cervical lymphadenopathy	0.027*	0.250 (0.073–0.851)	-2.28	0.009*	0.102 (0.018–0.571)
Platelet nadir (10^3^/μL)	0.025*	0.997 (0.994–1.0)	-0.005	0.173	0.995 (0.987–1.002)
Peak ALT (IU/L)	0.151	1.04 (0.985–1.01)			
Peak AST (IU/L)	0.127	1.02 (0.996–1.03)			
Peak creatinine (mg/dl)	0.09	5.77 (0.769–43.21)			

*ALT* alanine aminotransferase, *AST* aspartate aminotransferase

^*^P value ≤ 0.05 is considered statistically significant

**Fig. 2:**
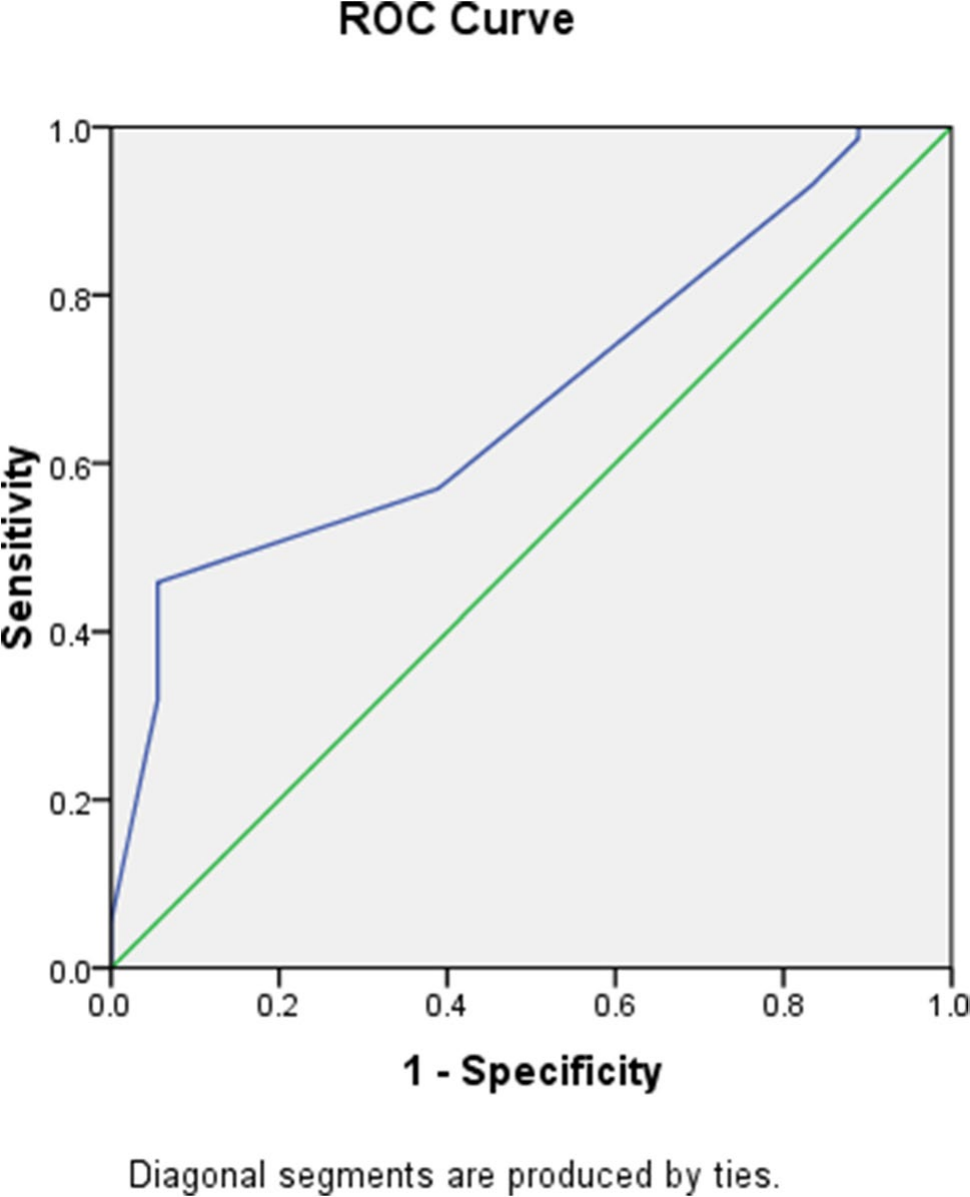
ROC curve of the calculated score in prediction of MIS-C from Kawasaki disease

Significant variables were assigned cut-off and scoring points based on the *β* coefficient (Table 5). These variables were utilized to generate a scoring model that yielded an AUC of 0.70 (95% CI 0.55–0.80). At the cut-off of 4 or more points, the sensitivity, specificity, and accuracy were 57%, 85.4%, and 57.8%, respectively, to distinguish MIS-C from KD in the scoring model (Table 6) (Fig. 2). Table 7 displays the likelihood of a child having MIS-C compared with KD. A cut-off greater than or equal to 4 suggests > 99% probability that this patient has MIS-C not KD.

**Table 5:** Independent predictors distinguishing MIS-C from Kawasaki disease in scoring model

Independent predictors	OR (95%CI)	Score^#^
Abdominal pain	3.03 (0.573–16.06)	1
Vomiting	5.52 (1.07–28.49)	2
Cervical lymphadenopathy	0.102 (0.018–0.571)	3
Platelet nadir (10^3^/μL) (≤ 397.8)^b^	0.995 (0.987–1.002)	1

^#^Weighted score is based on *β* coefficient for each variable calculated from univariate analysis; total score = 7

^b^Cut-off point based on the ROC curve

**Table 6:** The diagnostic accuracy of calculated score in prediction of MIS-C from Kawasaki disease

AUC	95% CI		Cut-off	Sensitivity	Specificity	PPV	NPV	Accuracy
0.70	0.556	0.803	4	57	85.4	61.1	26.2	57.8

*AUC* area under the curve, *CI* confidence interval, *NPV* negative predictive value, *PPV* positive predictive value

**Table 7:** The estimated probability of MIS-C versus KD at the score and cut-off points

Score points*	Probability of MIS-C	Probability of KD
• Score ≤ 2 • Score 3 • Score ≥ 4^b^	37.3% 70.6% > 99%	62.7% 29.4% 0

^*^Calculation of the probability based on each score separately cannot be applied, as some categories are zero

^b^Cut-off of the diagnostic score calculated by ROC curve

## Discussion

Many patients with MIS-C meet the criteria for KD that can make initial diagnosis and treatment decisions challenging. Hence, understanding and analysis of the subtle differences in presentation and laboratory markers by this comparative study can help in discrimination between both disorders.

The findings of this report were similar to the results of previously published studies that comparing MIS-C with KD patients specifically the male predominance, older age of presentation, longer, and higher rate of ICU admission in MIS-C [9, 20, 31].

Our study confirmed the preceding results given that MIS-C was commonly associated with GI symptoms (abdominal pain and vomiting) than those with KD [32–35]. Moreover, we observed that cervical lymphadenopathy was more pronounced in MIS-C patients rather than KD children [31, 36].

As expected, respiratory involvement (dyspnea, crepitation, and pneumonia) was rarely seen in KD patients, whereas in MIS-C, it occurred in 20–80% of our patients in line with previous case series [12, 16]. Likewise, Feldstein et al. (2021) [37] and Sperotto et al. (2021) [26] observed cardiac complications of MIS-C are common, including myocardial dysfunction, valve involvement, and coronary artery dilatation. Besides, the high rate of hypotension and shock required inotropic support in severe MIS-C [19, 23, 25].

Neurological complications or acute kidney injuries are more frequently reported in MIS-C patients than KD [19]. On the other hand, KD had a higher rate of dermatologic symptoms than MIS-C [36]. We supported the clinical evidence already published in the literature, but no statistical significant difference observed between the two groups regarding these organs involvement attributed to the small sample size in both groups.

Compared to KD, patients with MIS-C had lower platelet count that provided the highest biomarker specificity in MIS-C discernment similar to Kaushik et al. (2020) [38] and Riphagen et al. (2020) [17] conclusions. This difference is due to the variation in pathogenesis; the pathogenesis of KD is mediated by inflammatory cells that recruit more platelets. However, in MIS‐C, the viral‐associated hyperinflammation induce bone marrow suppression [8, 39].

Furthermore, it was noted that MIS‐C patients had lower levels of leukocytes, absolute lymphocyte count, and hemoglobin than KD; nevertheless, we could not gather this dissimilarity which might be due to age and sex variability of these parameters or analytical issues that reduce the reliability of these results [40]. MIS‐C patients had higher levels of ALT, AST, and serum creatinine than KD patients comparable with previously published results [31]. Systemic inflammation in MIS-C can involve multiple organs, such as liver, kidney, or heart, that lead to their biomarker elevation [40].

Both MIS‐C and KD patients showed elevated inflammatory markers including CRP and serum ferritin attributed to hyperinflammatory response in children, although more raise of inflammatory markers may be observed in MIS‐C than KD patients [41, 42]. Our cohort exhibited elevation of these acute phase reactants in MIS-C cases, but failed to show significant difference from KD patients. Even more, we compare CRP cut-off > 99.55 mg/L as a guide to discriminate between MIS-C and KD as suggested by Ganguly et al. (2022) [43], but there was no difference between the two groups.

In regression analysis, abdominal pain, vomiting, cervical lymphadenopathy, and platelet nadir were detected as the significant predictors of MIS-C from KD. These variables were utilized to generate a scoring model that yielded an AUC of 0.70 (95% CI 0.55–0.80), and the total score was 7 when all criteria added together.

This score is overlapping with the criteria recommended by Kostik et al. (2021) [31], and Godfred-Cato et al. (2022) [44] who developed a diagnostic score to distinguish MIS-C from KD. In these scores, age > 5 years, GI involvement, headache, pericardial effusion, D-dimer > 607 ng/ml, low platelets, elevated CRP, and the absence of rash or mucocutaneous lesions were the main predictors of MIS-C. The sum of these scores provided a high probability of MIS-C rather than KD.

Nevertheless, their KD patients were a historic cohort compared with newly diagnosed patients with MIS-C. Besides, Godfred-Cato et al. [44] differentiated MIS-C from multiple mimicking conditions like COVID-19, KD, and TSS with much diversity of patient data analysis. Their retrospective data gathering might be associated with some missing information. They included pericardial effusion as an ECHO finding that would not be readily available in the outpatient rooms. In addition, the Kostik’s score [31] incorporated D-dimer, which might not be accessible in some settings.

The emphasis of our observation relies on the fact that our KD patients were recruited during the same period of MIS-C enrolment, which reflects the real practice with a standard patient’s definition. It is a timely simple score that is based on initial clinical characteristics of patients and first tier universally available and routinely ordered investigations at all centers without the need for more specialized tests such as troponin, B-type natriuretic peptide, waiting ECHO, or other imaging modalities. These features making our score an effective differentiating tool at the point of preliminary evaluation before more costly or unavailable testing is ordered.

Our report suggests a cut-off of four in the scoring model to provide a simplified tool to distinguish MIS-C from KD. Nevertheless, more stringent studies with prospective analysis on larger series are critical to validate the proposed scoring model before practical application, as this score is not intended to be diagnostic criteria for MIS-C. In addition, our study’s limitations are that there were some missing data such as cardiac biomarkers, coagulable state, and small sample size. Future external validation with a power calculation using different sets of cases can improve its application, and we suggested that about 100 KD and 400 MIS-C patients are needed to get a clinically valuable scoring system.

## Conclusion

Children with MIS-C are more likely to have GI symptoms, cervical lymphadenopathy, and respiratory involvement. Moreover, elevated liver enzymes and lower platelet count are pronounced in MIS-C. This study constructed a prediction model for differentiation of MIS-C from KD based on clinical and laboratory profiles. This model will be valuable to guide clinicians in the treatment decisions.

## Data Availability

All data generated during this study are in this published article.
